# Predicting tolerability of high-dose fentanyl buccal tablets in cancer patients

**DOI:** 10.1371/journal.pone.0280212

**Published:** 2023-01-06

**Authors:** Mi-Young Kwon, Mi-Yeon Lee, Yun Jae Han, Sung Hyun Lee, Eo Jin Kim, Songyi Park, Yun‑Gyoo Lee, Dong-Hoe Koo

**Affiliations:** 1 Department of Anesthesiology and Pain Medicine, National Medical Center, Seoul, Korea; 2 Division of Biostatistics, Department of R&D Management, Kangbuk Samsung Hospital, Sungkyunkwan University School of Medicine, Seoul, Korea; 3 Department of Anesthesiology and Pain Medicine, Kangbuk Samsung Hospital, Sungkyunkwan University School of Medicine, Seoul, Korea; 4 Division of Hematology/Oncology, Department of Internal Medicine, Kangbuk Samsung Hospital, Sungkyunkwan University School of Medicine, Seoul, Korea; Bahauddin Zakariya University, PAKISTAN

## Abstract

**Background & aims:**

Fentanyl buccal tablets (FBTs) are a rapid-onset opioid indicated for breakthrough cancer pain (BTcP) and FBT titration is needed to optimize BTcP management. We aimed to predict which patients could tolerate a high dose of FBT (400 μg or more at a time).

**Methods:**

A retrospective analysis was performed to assess the final FBT dose. The final FBT doses were compared according to the clinical features. The prediction accuracy of patients tolerant of 400 μg or higher FBT was compared using the area under the receiver operating characteristic (ROC) curves. A risk scoring model based on the odds ratio (OR) was developed from the final multivariable model, and patients were assigned into two groups: low tolerance (0–1 point) and high tolerance (2–3 points).

**Results:**

Among 131 patients, the most frequently effective dose of FBT was 200 μg (54%), followed by 100 μg (30%). The median value of morphine equivalent daily doses (MEDD) was 60 mg/day, and the most common daily use was 3–4 times/day. In multivariable analysis, male sex, younger age, and use of FBTs three or more times per day were independently associated with high-dose FBT. According to the risk scoring model, the patients with a final FBT of 400 μg or higher were significantly more in the high tolerance group (17%) compared to the low tolerance group (3%; p = 0.023)

**Conclusions:**

According to the dose relationship between the final FBT dose and the clinical features, three factors (sex, age, daily use of FBT) were independently associated with the final dose of FBT. Our risk score model could help predict tolerance to high-dose FBT and guide the titration plan for BTcP.

## Introduction

Pain control is an essential part of cancer patient care [1]. Since the concept of breakthrough pain (BTcP), defined as a transitory increment of pain intensity in a setting of stable chronic pain managed with opioid drugs, was introduced in the 1990s [2], immediate-release opioids have been developed and are used for dealing with BTcP [3]. Furthermore, the rapid onset opioid (ROO) formulation was developed and produced in various fentanyl formulations [4–6]. These ROOs with fast action time have contributed dramatically to controlling the BTcP in cancer patients in clinical practice [7].

Since ROO formulations show a rapid action time, when an overdose is continuously administered, adverse events or drug misuse can be induced [8]. On the other hand, when a final effective dose of ROOs is not reached due to slow titration, they can result in poor satisfaction, non-compliance, and even failure to achieve adequate pain control [9, 10]. Physicians should consider seeking the final effective dose by fast titration to control pain appropriately and reduce the risk of aberrant behavior [11]. However, among the cancer pain management guideline, recommendations for using ROO formulations are lacking [12].

We assumed that, if physicians recognized the characteristics of patients who could tolerate a higher final effective dose (400 μg or more) while prescribing the most commonly used ROO, fentanyl buccal tablets (FBTs) [13], a rapid titration plan could be established, increasing patient compliance. Therefore, this study was designed to investigate the characteristics of patients with high final doses of FBTs (400 μg or more at a time) and to predict which patients could tolerate the high FBT dose.

## Materials and methods

### Patients and data collection

We retrospectively reviewed the medical records of patients with cancer that were treated with FBTs for BTcP between September 2014 and December 2018 at Kangbuk Samsung Hospital (Seoul, Korea). Patients were eligible for this study if they were 19 years of age or older, with histologically documented malignancy, if they received FBTs for more than two weeks with around-the-clock (ATC) opioids. The final FBT dose was assessed from medical records where physicians assessed successful pain relief and adverse events for the FBT dosage administered. For dose titration of FBT, the dose was continuously increased for subsequent episodes when BTcP was considered unsatisfactorily controlled, from 100 μg up to 200, 400, and 800 μg, to achieve the effective dose [14]. We defined the high dose of FBT as 400 μg or more FBTs at a time. Body surface area (BSA) was calculated by the Mosteller formula [15]. The BSA was categorized into 1) < 1.4 m^2^, 2) 1.4–1.69 m^2^, and 3) ≥1.7 m^2^. Baseline ATC opioids were converted to oral morphine equivalent daily dose (MEDD), and MEDD was categorized into 1) < 60 mg/day, 2) 60–89 mg/day, and 3) ≥90 mg/day. This study was approved by the Institutional Review Board (IRB) of Kangbuk Samsung Hospital (KBSMC IRB. 2019-07-016). Our analysis was a retrospective design using fully anonymized data, so the IRB waived the requirement for informed consent.

### Statistical analyses

Demographic and clinical variables were collected, and descriptive statistical analysis of relevant variables was performed to obtain number, percentage, mean, standard deviation (SD), and median and interquartile range (IQR). The final FBT doses were compared according to the patient distribution and clinical features to assess the association between the high final FBT dose and clinical features using chi-square analysis or Fisher exact test. The prediction accuracy of patients tolerant of 400 μg or higher FBT was compared using the area under the receiver operating characteristic (ROC) curves [AUC], which used the predicted values (C-statistics) of logistic regression analysis. A risk scoring model based on the odds ratio (OR) was developed from the final multivariable model, with one point scored for OR >5 and zero points scored for OR < 5. Based on the sum of scores, patients were assigned into two groups: low tolerance group or high tolerance group to high FBT. A two-sided *P-*value < 0.05 was considered statistically significant, and 95% CIs were calculated. All statistical analyses were performed using R language (R Core Team, R Foundation for Statistical Computing, Vienna, Austria) and the Statistical Package for the Social Sciences version 25.0 software program (IBM Corporation, Armonk, NY, USA).

## Results

### Patient characteristics

A total of 131 patients was eligible for this analysis. The baseline characteristics of patients are summarized in Table 1. Males represented 62% of the patients, and the median age was 62 years. The mean body height and weight were 162.1 cm and 55.9 kg, respectively. The mean BSA and BMI were 1.58 m^2^ and 21.2 kg/m^2^, respectively. The most common tumors were colorectal cancers (34%), and almost all patients (n = 124, 95%) had stage IV disease. The median value of oral MEDD was 60 mg/day (range, 15–960 mg), and 41 patients (31%) had received 90 mg or more of oral MEDD. Overall, the final dose of FBT was 200 μg in 71 patients (54%), 100 μg in 39 patients (30%), 400 μg in 17 patients (13%), and 800 μg in 4 patients (3%). The number of daily FBTs used was 3–4 times/day in 91 patients (70%), 1–2 times/day in 33 patients (25%), and ≥5 times/day in 7 patients (5%). In addition, 30 patients (23%) had received concurrent NSAIDs.

**Table 1:** Patient characteristics (n = 131).

Variables	Value
**Sex**	
** Male**	81 (61.8)
** Female**	50 (38.2)
**Age (years)**	62 (32–82)
** < 60**	61 (46.6)
** 60–69**	35 (26.7)
** ≥ 70**	35 (26.7)
**Height (cm)**	162.1±8.8
**Weight (kg)**	55.9±10.2
**BSA (m** ^**2**^ **)**	1.58±0.16
**BMI (kg/m** ^**2**^ **)**	21.2±3.22
**Primary tumor site**	
** Gastric cancer**	35 (26.7)
** Hepatobiliary cancer**	18 (13.7)
** Pancreatic cancer**	23 (17.6)
** Colorectal cancer**	45 (34.4)
** Others**	10 (7.6)
**Stage**	
** II**	7 (5.3)
** IV**	124 (94.7)
**MEDD (mg/day)**	60 (15–960)
** < 60**	60 (45.8)
** 60–89**	30 (22.9)
** ≥ 90**	41 (31.3)
**Frequency of FBT use**	3 (1–12)
** 2 or less/day**	33 (25.2)
** 3-4/day**	91 (69.5)
** 5 or more/day**	7 (5.3)
**Concurrent NSAIDs (Received)**	30 (22.9)

Values are expressed as number (percentage), median (range), or mean ± standard deviation.

BSA, body surface area; BMI, body mass index; MEDD, oral morphine equivalent daily dose; FBT, fentanyl buccal tablet; NSAIDs, non-steroidal anti-inflammatory drugs.

### Associations between final FBT dose and clinical factors

**Fig 1:**
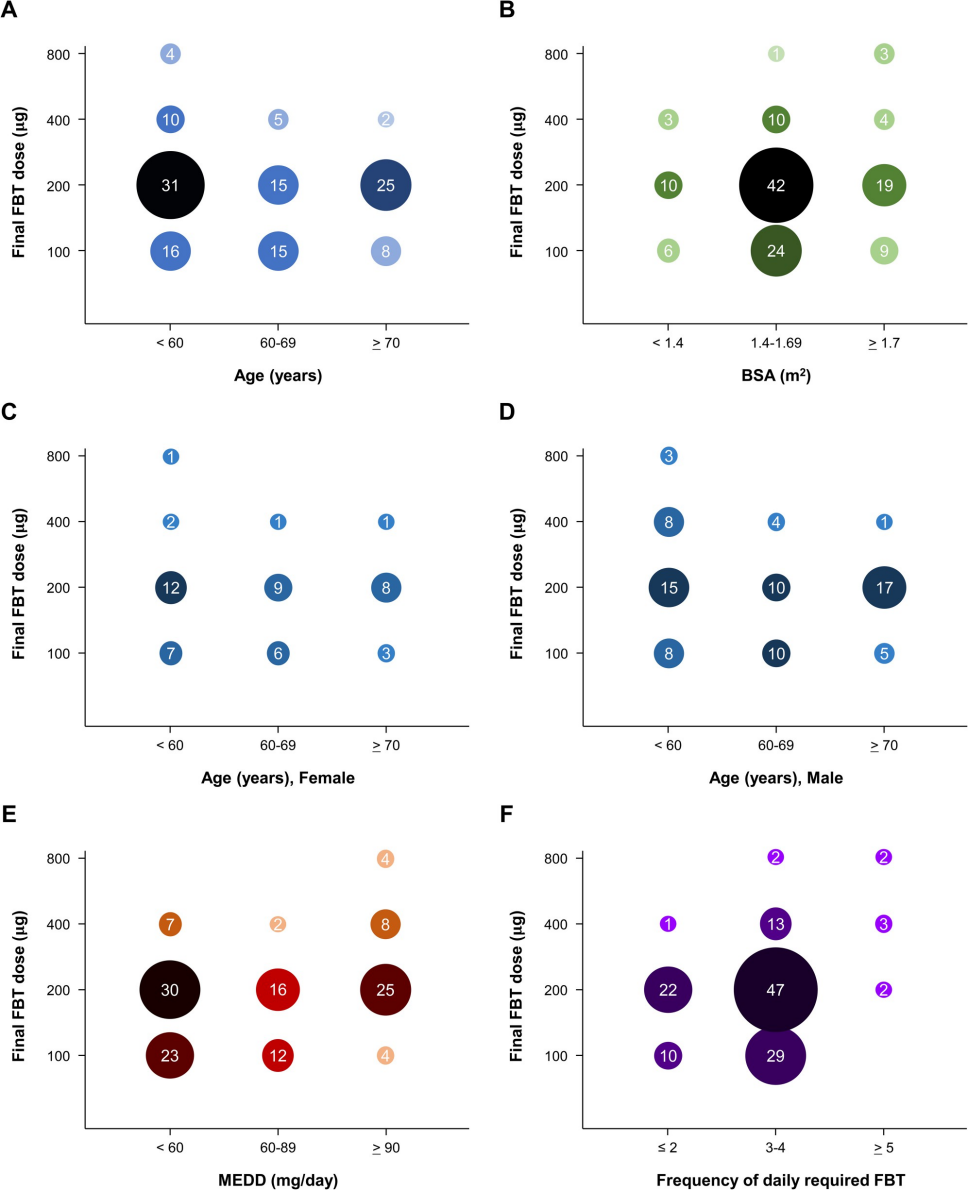
Patient distribution between final dose of fentanyl buccal tablet (FBT) and (a) age, (b) body surface area (BSA), (c) age in females, (d) age in males, (e) oral morphine equivalent daily dose (MEDD), and (f) frequency of daily required FBT use. The number inside each circle indicates the patient count, and the size of the circle correlates with the number of patients.

The final FBT doses were analyzed according to clinical characteristics, and the associations between FBT dose and characteristics were evaluated (Table 2; Fig 1). The final FBT dose had an inverse relationship with patient age (*P* = 0.055); the younger patients had a higher final FBT. Higher MEDD and more frequent FBT daily use were associated with significant increases in final FBT dose (*P* = 0.003; *P* < 0.001, respectively). There was no association between final FBT and any physique index: BSA (*P* = 0.506), BMI (*P* = 0.769), and weight (*P* = 0.159).

**Table 2:** Patient distribution between final fentanyl buccal tablet (FBT) dose and clinical factors.

Variables	Final FBT dose				*P*-value
	100 μg	200 μg	400 μg	800 μg	
**Sex**					0.534
** Male**	23 (28.4)	42 (51.9)	13 (16.0)	3 (3.7)	
** Female**	16 (32.0)	29 (58.0)	4 (8.0)	1 (2.0)	
**Age (years)**					0.055
** < 60**	16 (26.2)	31 (50.8)	10 (16.4)	4 (6.6)	
** 60–69**	15 (42.9)	15 (42.9)	5 (14.3)	-	
** > 70**	8 (22.9)	25 (71.4)	2 (5.7)	-	
**Weight (kg)**					0.159
** < 50**	11 (31.4)	18 (51.4)	5 (14.3)	1 (2.9)	
** 50–64.9**	22 (31.0)	40 (56.3)	9 (12.7)	-	
** > 65**	6 (24.0)	13 (52.0)	3 (12.0)	3 (12.0)	
**BSA (m** ^**2**^ **)**					0.506
** < 1.4**	6 (31.6)	10 (52.6)	3 (18.5)	-	
** 1.4–1.69**	24 (31.2)	42 (54.5)	10 (13.0)	1 (1.3)	
** > 1.7**	9 (25.7)	19 (54.3)	4 (11.4)	3 (8.6)	
**BMI (kg/m** ^**2**^ **)**					0.769
** < 18.5**	8 (36.4)	10 (45.5)	4 (18.2)	-	
** 18.5–22.9**	23 (30.3)	42 (55.3)	9 (11.8)	2 (2.6)	
** > 23.0**	8 (24.2)	19 (57.6)	4 (12.1)	2 (6.1)	
**MEDD (mg/day)**					0.003
** < 60**	23 (38.3)	30 (50.0)	7 (11.7)	-	
** 60–89**	12 (40.0)	16 (53.3)	2 (6.7)	-	
** > 90**	4 (9.8)	25 (61.0)	8 (19.5)	4 (9.8)	
**Frequency of FBT use**					< 0.001
** 2 or less/day**	10 (30.3)	22 (66.7)	1 (3.0)	-	
** 3-4/day**	29 (31.9)	47 (51.6)	13 (14.3)	2 (2.2)	
** 5 or more/day**	-	2 (28.6)	3 (42.9)	2 (28.6)	

Values are expressed as number (percentage).

BMI, body mass index; BSA, body surface area; MEDD, oral morphine equivalent daily dose; FBT, fentanyl buccal tablet.

### Comparison of prediction and multivariable analysis for tolerance of 400 μg of FBT

**Fig 2:**
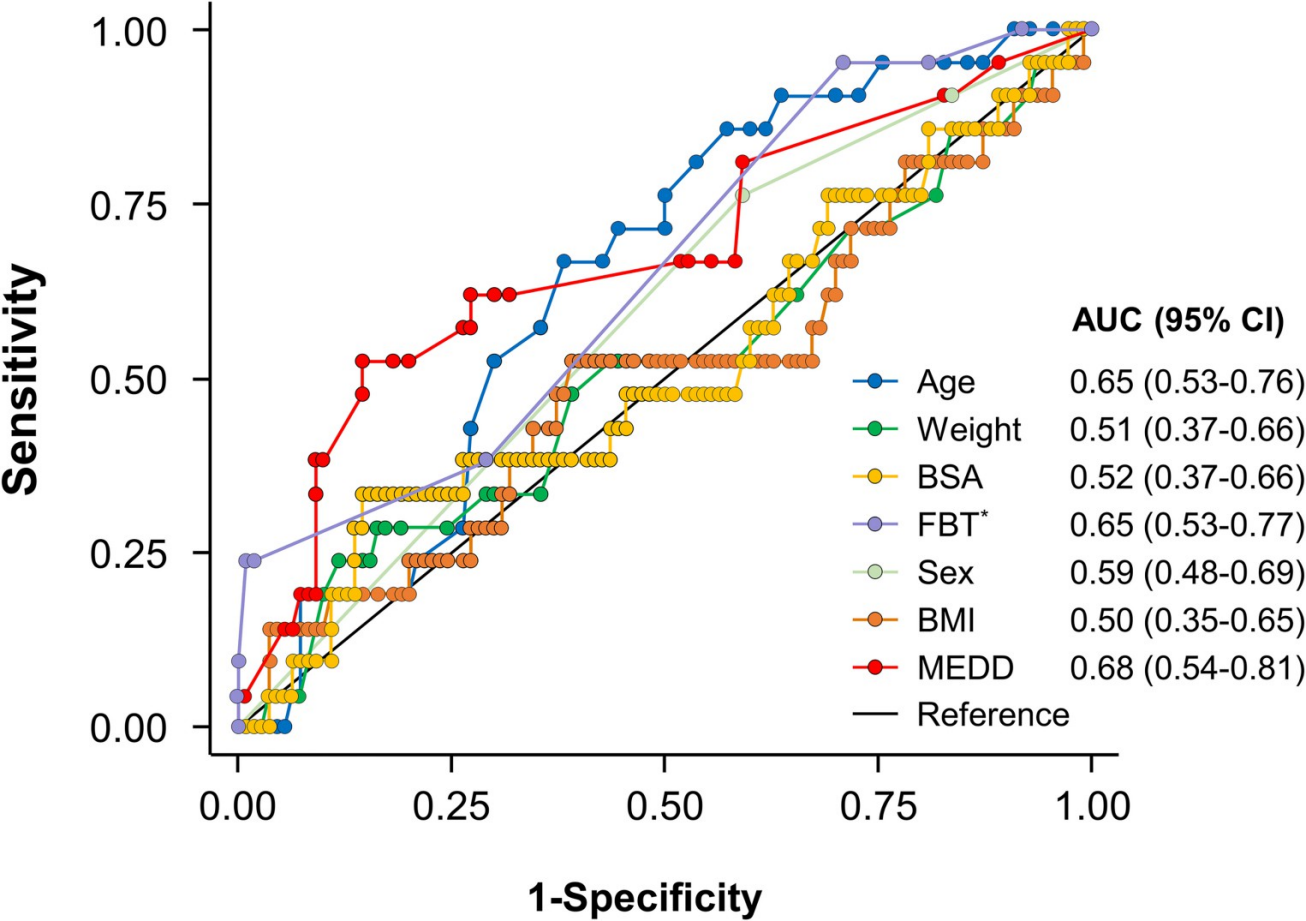
Receiver operating characteristic (ROC) curves for prediction of tolerability of high-dose fentanyl buccal tablet (FBT). BMI, body mass index; BSA, body surface area; MEDD, oral morphine equivalent daily dose; FBT, frequency of fentanyl buccal tablets required daily.

ROC curve analysis was performed to compare the predictability of a final high dose FBT according to the clinical features. The ROC curve analysis showed similar AUCs of three factors; age, MEDD, and frequency of daily FBT use (0.65 [95% CI: 0.53–0.76], 0.68 [95% CI: 0.54–0.81] and 0.65 [95% CI: 0.53–0.77], respectively [Fig 2]). When the predictive accuracy was compared among the body physique metrics after adjustment for age, sex, MEDD, and frequency of daily FBT, BSA had a slightly larger AUC (0.88; 95% CI: 0.81–0.95) compared to weight and BMI (0.85 [95% CI: 0.76–0.94], and 0.84 [95% CI: 0.75–0.94], respectively [S1 Fig]).

Therefore, multivariable analysis was performed using sex, age, BSA, MEDD, and frequency of daily FBT use. Among them, male sex (*P* = 0.031), younger age (*P* = 0.019), and frequent use of FBT per day (*P* = 0.001) were independently associated with final FBT 400 μg or higher. Risk scores were assigned based on the OR from the final multivariable model (Table 3), with one point awarded for OR >5.0 (male; < 70 years; FBT use three or more times daily). Based on scores, patients were assigned into two groups: low tolerance to higher FBT (0–1 point) and high tolerance (2–3 points). According to risk scoring groups, only one of 33 low-tolerance patients (3%) had a 400 μg final FBT, and most patients with a final FBT 400 μg or higher were classified into the high tolerance group (*P* = 0.023; Table 4).

**Table 3:** Multivariable analysis for tolerability of fentanyl buccal tablet (FBT) 400 μg or higher (final FBT, 400/800 μg vs. 100/200 μg).

Variables	OR (95% CI)	*P*-value	Scores^a^
**Sex**			
** Female**	1 (reference)	-	0
** Male**	5.41 (1.16–25.16)	0.031	1
**Age (years)**			
** > 70**	1 (reference)	0.078	0
** 60–69**	7.78 (0.91–66.37)	0.024	1
** < 60**	9.83 (1.35–71.74)	0.019	1
**BSA (m** ^**2**^ **)**			
** < 1.4**	1 (reference)	0.218	-
** 1.4–1.69**	0.28 (0.04–1.86)	0.188	-
** > 1.7**	0.13 (0.01–1.29)	0.081	-
**MEDD (mg/day)**			
** < 60**	1 (reference)	0.019	-
** 60–89**	0.21 (0.03–1.48)	0.117	-
** > 90**	3.11 (0.89–10.89)	0.076	-
**Frequency of FBT use**			
** 2 or less/day**	1 (reference)	0.002	0
** 3-4/day**	6.60 (0.67–65.08)	0.106	1
** 5 or more/day**	180.67 (8.48–3848.76)	0.001	1

^a^ Scores were graded as 1 or 0 based on multivariable logistic regression.

BMI, body mass index; BSA, body surface area; CI, confidence interval; FBT, fentanyl buccal tablet; MEDD, oral morphine equivalent daily dose; OR, odds ratio.

**Table 4:** Final fentanyl buccal tablet (FBT) dose according to risk scores based on odds ratio (OR) from the final multivariate model.

	Sum of score	Final FBT dose, n (%)				*P*-value
		100 μg	200 μg	400 μg	800 μg	
**Risk group**						0.023
** Low tolerance**	0–1	7 (21.2)	25 (75.8)	1 (3.0)	-	
** High tolerance**	2–3	32 (32.7)	46 (46.9)	16 (16.3)	4 (4.1)	

FBT, fentanyl buccal tablet.

## Discussion

This study evaluated the association between patient’s characteristics and final high dose FBT to predict which patients could tolerate the high FBT dose. A risk score model was developed using three factors (sex, age, frequency of daily required FBT) independently associated with high final dose of FBT. If the sum of the score was two or more, the patient is suggested to be tolerant of high-dose FBT.

### Recent studies and guidelines for BTcP control

BTcP should be treated with agents that have a quick onset and short duration with a rescue dose of 10% to 20% of the total daily dose of the maintenance opioid [14]. FBT formulation has become a standard of care because it has advantages suitable for BTcP control with fast onset time and short action time [8, 16–18]. No clinical characteristics were associated with a successful titration or final effective FBT dose [17, 18]. Therefore, the current guidelines have stated that FBT should be started on the lowest dose of the formulation and titrated to an effective dose [14]. Recently, however, several studies suggested that a titration process starting with the lowest doses of FBT did not have any advantage, and a higher starting dose (more than 100 μg) of FBT proportional to the basal opioid regimen seemed to be safe and effective [19–22].

### Difficulties in BTcP control with rescue medication

However, it is not easy to control BTcP appropriately in cancer patients, especially in outpatient clinics. A recent survey reported that physicians had difficulty managing BTcP in cancer patients with their rescue medications [23]. When an insufficiently low dose is used, the FBT cannot adequately control the patient’s pain. Approximately 30% of patients with adequately controlled background pain complained of poor BTcP control even in the in-patient setting [24]. On the other hand, if the FBT overdoses, patients may experience adverse effects such as nausea/vomiting and dizziness and then avoid the medication [25]. A titration plan to achieve a final effective dose of FBT also can be helpful since dose escalation without proper titration results in FBT overdose and poor BTcP control [18]. Therefore, to maintain the quality of life in cancer patients, it is necessary to prescribe the FBT safely and effectively according to the unique character of the ROO [23].

### Novel prediction model for tolerability of high-dose FBT

During outpatient FBT prescription for BTcP, an effective dose is decided through step-by-step titration [14, 21]. Some patients require caution in dose escalation due to the possible adverse effects, while others are tolerant of high-dose FBT and can achieve more aggressive and rapid titrations [25]. This study found that patients who were male, younger, or used FBTs more frequently tended to receive higher doses of FBTs. The simple risk scoring model was developed and scored with one point each for male sex, age < 70 years, or use of FBTs three or more times per day. Patients with two or more points were classified as a high tolerance group. This proposed model can help physicians predict who can tolerate high-dose FBT (more than 400 μg) since these three factors (sex, age, and daily use of FBT) are easily obtained from clinical practice. On the other hand, different types of body measurement, such as body weight, BSA, and BMI, did not correlate with FBT dose. This is similar to the results not found in previous studies using body measurements to predict BTcP [24].

### Limitations of this study

Our study had several limitations that are inherent to the retrospective design and analysis. There were a small number of patients, and all were treated at a single institution in Korea. No information about cancer disease and background pain, defined as an average pain intensity score < 4/10 on the NRS, was available [24]. Also, data regarding BTcP intensity at and after initial titration with FBTs were not available. Finally, the proposed predicting model was not validated. Despite these limitations, this analysis provides insight into physicians’ practice in considering patient tolerance of a high-dose FBT. Furthermore, some patients are intolerant or require a low-dose FBT, so escalation of the dose must be performed carefully when prescribing ROO drugs such as FBT. Since social reports about side effects such as opioid dependence or abuse have continued to grow, physicians must prescribe opioids carefully and take into consideration patient characteristics as well as clinical conditions and comorbidities [26].

In conclusion, we identified three factors readily measured in clinical practice and predictive of tolerance to high-dose FBT in cancer patients. Our prediction model is based on a scoring system and can classify patients into low and high tolerance groups. One point each is assigned for male sex, age younger than 70 years, or FBT use three or more times per day. Patients with two or three points can be considered to have high tolerance to FBT, and high-dose FBT can be applied after short titration periods. This model could help predict tolerance to high-dose FBT and guide the titration plan for BTcP.

## Supporting information

S1 Fig Receiver operating characteristic (ROC) curves for prediction of tolerability of high-dose fentanyl buccal tablets after adjustment of age, sex, frequency of daily fentanyl buccal tablet (FBT) use, and oral morphine equivalent daily dose (MEDD). BMI, body mass index; BSA, body surface area.
(TIF)
