# Supplementary figures and images for: Predicting tolerability of high-dose fentanyl buccal tablets in cancer patients
Source: PLoS One. 2023 Jan 6;18(1):e0280212. doi: 10.1371/journal.pone.0280212 (PMC9821425; doi:10.1371/journal.pone.0280212)

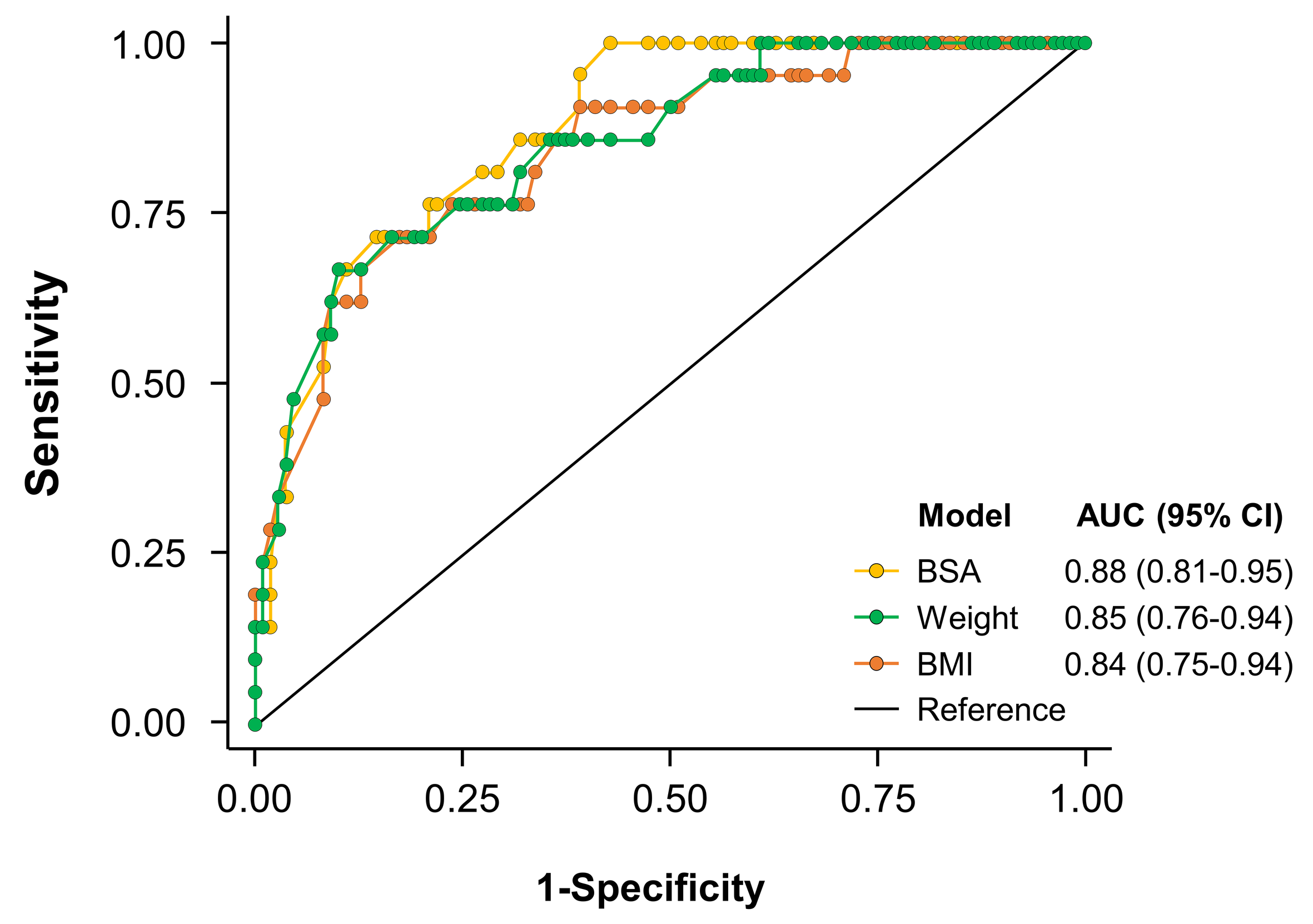

Supplement: S1 Fig — BMI, body mass index; BSA, body surface area. (TIF) [file pone.0280212.s001.tif]
